# Secundiparas following a failed vacuum delivery—factors associated with a successful vaginal delivery: a historical prospective cohort

**DOI:** 10.1186/s12884-022-05151-7

**Published:** 2022-11-02

**Authors:** Orna Reichman, Zvi Ehrlich, Ramy Suday, Hen Sela, Gila Gold, Arnon Samueloff, Sorina Grisaru-Granovsky

**Affiliations:** Department of Obstetrics and Gynaecology, Shaare Zedek Medical Centre, Hebrew University, Jerusalem, Israel

**Keywords:** Failed vacuum-delivery, TOLAC, VBAC, Birthweight

## Abstract

**Background:**

Few studies have focused on the delivery subsequent to a failed vacuum delivery (failed-VD) in secundiparas. The objective of the current study was to examine the factors associated with a vaginal delivery following a failed-VD.

**Methods:**

An historical prospective cohort. Obstetric characteristics of secundiparas who underwent a planned caesarean delivery (CD) were compared to those who elected a trial of labour (TOLAC) at single medical-centre, throughout 2006–2019. The latter were further analysed to study for factures associated with successful vaginal birth (VBAC).

**Results:**

Among the 115 secundiparas included, 89 (77%) underwent TOLAC. Compared to women who underwent an elective CD, those who underwent TOLAC were younger by a mean of 4 years, were more likely to have conceived spontaneously, and had a more advanced gestation by a mean of 10 days. VBAC was achieved in 62 women (70%). New-borns of women with VBAC had in average a lower birth weight compared to those with failed TOLAC, (-)195 g ± 396 g versus ( +)197 g ± 454 g respectively, *P* < 0.01. Having a higher neonatal birthweight at P2 by increments of 500 g, 400 g or 300 g was associated with a failed TOLAC; OR of 9.7 (95%CI; 2.3, 40.0), 11.5 (95%CI; 2.8, 46.7) and 4.5 (95%CI; 1.4, 13.9), respectively.

**Conclusions:**

Among secundiparas with a previous CD due to a failed-VD, the absolute difference of neonatal BW was found to be significantly associated with achieving VBAC.

## Background

Assisted vacuum delivery is a common obstetric intervention, practiced in most medical centres, with a prevalence of 3–14% [1–3]. Potential neonatal and maternal complications associated with this mode of delivery require that the procedure be performed by experienced obstetricians, only when medically indicated, and when the position of the foetal head, as well as the cervical conditions, are appropriate [4]. The reported rate of emergency caesarean delivery (CD) secondary to failed vacuum delivery (failed-VD) is consistent among studies and ranges between 4–6% [5–8]. Although long-term neurological and haematological complications were not found to be associated with a failed-VD [9, 10], short-term adverse neonatal outcomes and maternal complications are well described [11]. Factors known to be associated with failed-VD include, nulliparity, macrosomia, induction of labour, hypotonic uterine contractions, and persistent occiput posterior presentation (POP) [5–8]. Few studies have focused on the delivery subsequent to the failed-VD [12–14]. Two of these studies, which included 206 women of various parity, searched for factors that could assist in decision-making prior to attempting a trial of labour after CD, secondary to a failed-VD in the previous delivery (TOLAC failed-VD) [12, 13]. Findings were inconsistent between the studies, with only one study identifying risk factors from the index failed-VD associated with a failed TOLAC in the subsequent delivery. These factors included prolonged second stage, non-POP, and lower birthweight (BW) of the new-born [12]. Due to the limited literature available, the primary objective of the current study was to examine factors associated with a successful TOLAC in secundiparas (parity = 2) following a failed-VD. Specifically, we hypothesized, based on clinical experience, that the difference between the BW of the new-borns at P1 and P2, with each woman serving as her own control, would be a significant factor associated with a successful TOLAC failed-VD.

## Methods

### Study design

A historical prospective cohort study was performed in a single large tertiary university hospital, Shaare Zedek Medical Centre (SZMC), which manages approximately 10% of the annual national deliveries, 14,500 deliveries per year. The medical centre mainly serves religious Orthodox Jews and Muslims, a population which tends to have large families. Twenty five percent of parturients are nulliparous and approximately 18% are grand multiparous ≥ 6 deliveries. Over 95% of deliveries are funded by national public insurance, managed by residents and midwives and supervised by senior obstetricians at all times.

TOLAC is a common practice, attempted by 80% of suitable women at the study facility. Women are eligible for TOLAC after confirmation of a single previous low-segment transverse uterine incision (irrespective of the number of layers of the uterine closure) and a foetal weight estimation below 4200 g within a week of admission, either by clinical assessment or ultrasound examination. After an explanation of the TOLAC protocol and potential complications to the woman by the admitting obstetrician, verbal consent is documented in the medical record.

### Study population

All secundiparas (parity 2), at term, with a history of CD due to a failed-VD in their previous delivery, who delivered at SZMC throughout January 2006- December 2019 were identified by screening the electronic medical database for 'failed- VD', as encoded by the International Classification of Diseases (660.7) at P1. Secundiparas who delivered P1 at another facility were identified by a computerized search for "previous failed vacuum" in the electronic medical record (EMR). Women with previous vaginal deliveries, multiple pregnancies, and those who delivered elsewhere subsequent to the failed-VD were excluded.

### Data collection procedure

Data were extracted from a computerized database that was updated, in real-time, by midwives and obstetricians attending the labour and delivery. At least 50% of data fields are fixed and required to be completed before transferring the parturient to the postpartum floor. Data retrieved for both P1 and P2 included maternal age, gestational age at delivery and new-born birthweight (BW). In accordance with findings from previous studies, we obtained the length of the second stage and POP presentation of P1. Data retrieved from P2 included use of artificial reproductive treatment, interpregnancy interval (months), maternal gestational diabetes or hypertension, onset of labour (spontaneous, induction or planned CD), augmentation with oxytocin, presence of severe meconium, gender of the new-born, and mode of delivery. Head circumference (HC) was available only for the neonates born at SZMC. BW of the new-borns were studied as categorical and continuous variables: (1) absolute delta between P1and P2, calculated for each woman independently categorized into > 500 g, > 400 g and > 300 g and (2) mean delta compared between P1and P2.

### Data management and analysis

Data was validated by defining distributions and quantifying missing values. Obstetric characteristics are presented as proportions or means, for categorical or continuous variables, respectively, and stratified by a successful TOLAC. Statistical significance was defined by a two-sided *p* value ≤ 0.05 using the Chi-square test for categorical variables and Student t-test for continuous ones. Bivariate analysis was performed to evaluate if factors related to the index failed vacuum delivery, P1, are associated with a successful VBAC in the subsequent delivery (OR, 95% CI). A multivariate logistic regression modelling was to be performed if more than one factor was to be found associated with a successful TOLAC.

Sample size was estimated based on a previous publication, which showed that among women undergoing TOLAC failed-VD, with the BW of the new-born at P2 > P1, VBAC rate was 3.5% as opposed to a rate of 47% for failed TOLAC [12]. Hence, the calculated sample size required to show such a difference was 30 women (15 in each group). The large number of deliveries performed at the study site over the study period (over 200,000) was deemed sufficient to yield the sample size needed for the current study.

## Results

**Fig. 1:**
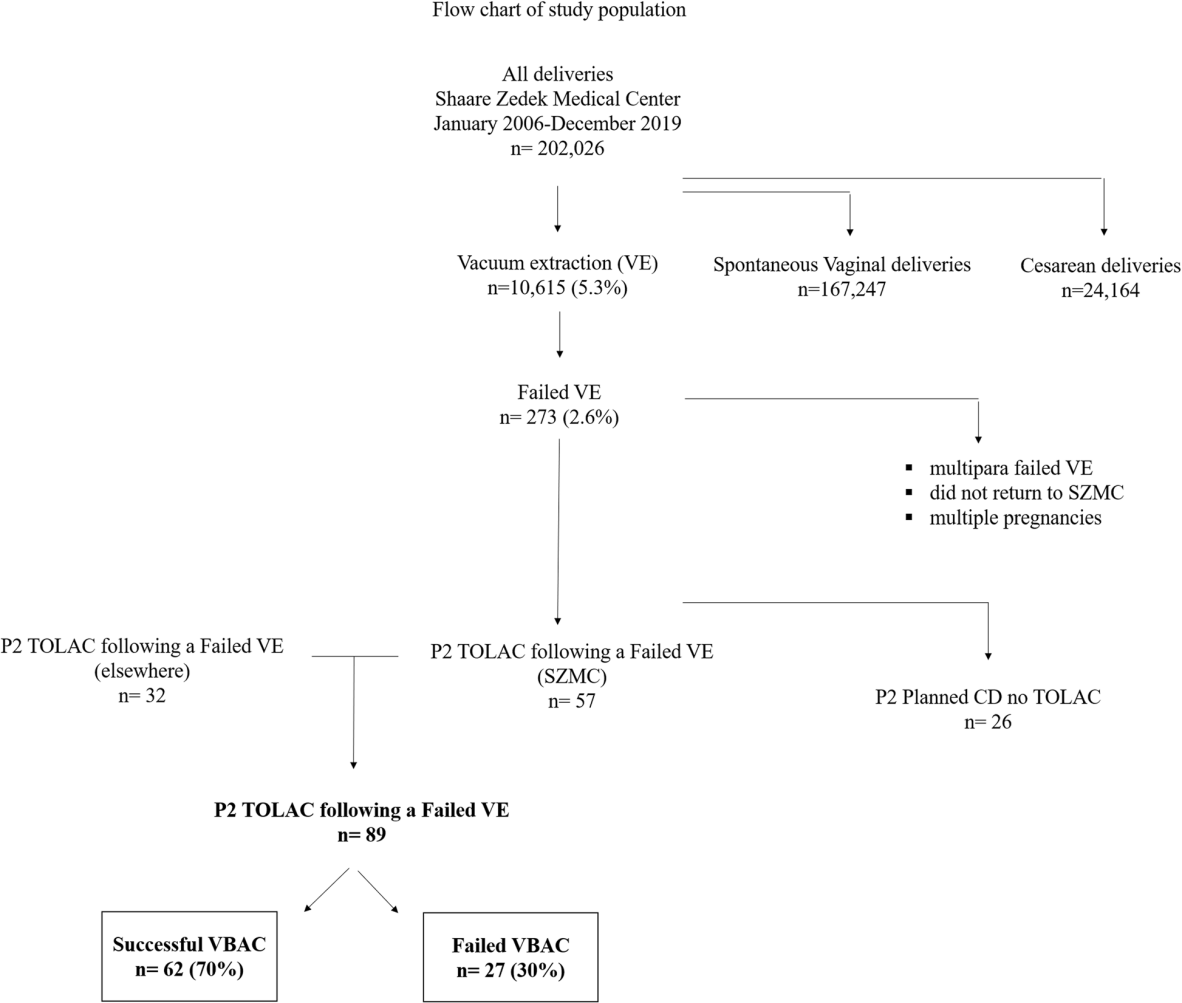
Flow chart of study population

During the 14 years of the study period, there were 202,026 deliveries of which 10,615 (5.3%) were instrumental vacuum deliveries. Failure followed by an emergency CD was reported in 273 (2.6%) of the vacuum deliveries. Among the 115 secundiparas (parity = 2) that met inclusion criteria, 89 (77%) underwent TOLAC (Fig. 1).

Women who underwent TOLAC were younger by a mean of four years, were more likely to have conceived spontaneously, and had advanced gestation by 10 days compared to those who elected for a planned CD, as seen in Table 1.

**Table 1:** Characteristics of secundiparas following a CD due to failed-VE, stratified by TOLAC versus planned CD

	TOLAC *n* = 89	planned caesarean *n* = 26	*P* value
**Factors at P1 (CD due to failed VE)**			
gestation at delivery (mean ± std)	40.1 ± 1.4	39.7 ± 1.4	0.26
indication for VE prolonged 2^nd^ stage N (%)	32 (56.1%)	9 (50%)	0.78
persistent occiput posterior N (%)	8 (13.8%)	2 (11.1%)	1.0
birthweight of new-born (mean ± std)	3395 g ± 363 g	3366 g ± 386 g	0.72
macrosomia (> 4000 g) N (%)	4 (4.8%)	2 (7.7%)	0.62
delivered elsewhere N (%)	32 (36.0%)	8 (30.8%)	0.81
**Factors at P2 (TOLAC)**			
maternal age (mean ± std)	27.4 ± 3.7	31.8 ± 5.7	< 0.01
artificial reproductive treatment N (%)	0	6 (30%)	< 0.01
interpregnancy interval, months (mean ± std)	29.2 ± 10.5	35.4 ± 17.7	0.07
gestational hypertension N (%)	0	0	
gestational diabetes N (%)	3 (3.6%)	0	1.0
gestation at delivery (mean ± std)	39.2 ± 1.8	37.6 ± 1.0	< 0.01
preterm delivery < 37 weeks N (%)	5 (5.6%)	1 (3.8%)	1.0
thick meconium N (%)	5 (6.8%)	0 (0%)	NA
new-born—male N (%)	44 (49.4%)	18 (69.2%)	0.12
macrosomia (> 4000 g)	4 (4.5%)	0	0.57
birthweight of new-born (mean ± std)	3300 g ± 466 g	3190 g ± 297 g	0.26
delta birthweight of new-born (P2-P1) (mean ± std)	-73.1 g ± 450 g	-175 g ± 385 g	0.30
delta birthweight of new-born > 500 g (P2 > P1) N (%)	12 (14.3%)	1 (3.8%)	0.29
delta birthweight of new-born > 300 g (P2 > P1) N (%)	17 (20.2%)	2 (7.7%)	0.23
delta birthweight of new-born > 500 g (P1 > P2) N (%)	18 (21.4%)	4 (15.5%)	0.58
delta birthweight of new-born > 300 g (P1 > P2) N (%)	28 (33.3%)	7 (27.9%)	0.63

*VBAC* Vaginal birth after caesarean, *TOLAC* Trial of labour after caesarean

VBAC was achieved in 62 women (70%). During the study period, the overall success rate at our facility for TOLAC among secundiparas, regardless of the indication for the previous CD, was 75% (2916/3881). Applying the χ^2^ test demonstrated no significant difference between the 70% success rate in secundiparas undergoing TOLAC subsequent to a failed-VD to the 75% success rate of TOLAC subsequent to CDs performed regardless of the indication for the previous CD, *p* = 0.23. P1 characteristics such as prolonged second stage, POP, macrosomia, BW of the new-born and delivery in another facility were not found to be associated with a VBAC (Table 2).

**Table 2:** Characteristics of secundiparas undergoing TOLAC following a failed-VE, stratified by a successful VBAC

	VBAC *n* = 62	Failed TOLAC *n* = 27	*P* value
**Factors at P1 (CD due to failed VE)**			
gestation at delivery (mean ± std)	40 ± 1.3	40 ± 1.7	0.48
indication for VE prolonged 2^nd^ stage N (%)	21 (55%)	11 (58%)	0.85
persistent occiput posterior N (%)	6 (16%)	2 (10%)	0.70
birthweight of new-born (mean ± std)	3429 g ± 340 g	3320 g ± 407 g	0.20
macrosomia (> 4000 g) N (%)	4 (7%)	0 (0%)	0.31
delivered elsewhere N (%)	25 (40%)	7 (26%)	0.23
**Factors at P2 (TOLAC)**			
maternal age (mean ± std)	27.4 ± 3.9	27.3 ± 3.5	0.92
artificial reproductive treatment N (%)	0	0	
interpregnancy interval (months) (mean ± std)	27.6 ± 9.1	32.3 ± 12.3	0.10
gestational hypertension	0	0	
gestational diabetes N (%)	1 (1.6%)	2 (7.4%)	0.22
gestation at delivery (mean ± std)	39 ± 1.4	38 ± 2.7	0.21
preterm delivery < 37 weeks N (%)	2 (3.2%)	3 (11.1%)	0.16
induction of labour N (%)	4 (6.5%)	1 (3.7)	1.0
augmentation with oxytocin N (%)	42 (68%)	16 (59%)	0.30
thick meconium N (%)	5 (8.2%)	0 (0%)	0.57
new-born—male N (%)	27 (44%)	17 (63%)	0.09
persistent occiput posterior N (%)	1 (1.6%)	2 (7.4%)	0.12
macrosomia (> 4000 g)	2 (3.2%)	2 (7.4%)	0.59
birthweight of new-born (mean ± std)	3247 g ± 352 g	3421 g ± 650 g	0.11
delta birthweight of new-born (mean ± std)	-195 g ± 396 g	197 g ± 454 g	< 0.01
delta birthweight of new-born > 500 g (P2 > P1)	3 (5%)	9 (35%)	< 0.01
delta birthweight of new-born > 400 g (P2 > P1)	3 (5%)	10 (39%)	< 0.01
delta birthweight of new-born > 300 g (P2 > P1)	7 (12%)	10 (39%)	< 0.01
delta birthweight of new-born > 500 g (P1 > P2)	16 (27.6%)	2 (7.7%)	0.04
delta birthweight of new-born > 400 g (P1 > P2)	21 (36.2%)	3 (11.5%)	0.03
delta birthweight of new-born > 300 g (P1 > P2)	24 (41.4%)	4 (15.4%)	0.02
head circumference cm (mean ± std)^a^	35.0 ± 1.3	34.4 ± 2.6	0.29
delta circumference mm P1 minus P2 (mean ± std) ^a^	3.4 ± 19.5	-2.9 ± 14.8	0.25
head circumference P2 > P1 by at least 10 mm	10 (28.6%)	8 (44.4%)	0.36

*VE* Vacuum Extraction, *VBAC* Vaginal birth after caesarean, *TOLAC* Trial of labour after caesarean

^a^data available for 53 women

The absolute difference in the BW of the new-born, comparing P1 to P2, was significantly associated with VBAC. New-borns of women with VBAC had in average a lower birth weight compared to those with failed TOLAC, (-)195 g ± 396 g versus ( +)197 g ± 454 g respectively, *P* < 0.01. Higher neonatal BW at P1 by 500 g, 400 g or 300 g was associated with a VBAC; OR of 4.5 (95%CI; 0.97, 21.6), 4.4 (95%CI; 1.2, 16.2) and 3.8 (95%CI; 1.2, 12.7) respectively. In contrast, higher neonatal birthweight at P2 (TOLAC failed-VD) by 500 g, 400 g or 300 g were associated with failed TOLAC; OR of 9.7 (95%CI; 2.3, 40.0), 11.5 (95%CI; 2.8, 46.7) and 4.5 (95%CI; 1.4, 13.9), respectively. Of the 89 secundiparas who underwent TOLAC, 52 (58.4%) had HC documented at both P1 and P2. As noted in Table 2, there was an absolute HC difference of at least 10 mm P2 > P1, calculated per woman, for 10/62 (16%) of the VBAC women compared to 8/27 (44.4%) of the failed TOLAC (*p* = 0.36). Artificial reproductive treatments, pregnancy induced hypertension, gestation diabetes, POP, and macrosomia were uncommon among the study group, < 4%. Multivariate analysis was not performed given that only one factor, "the absolute difference in neonatal BW", was found to be significantly associated with VBAC.

## Discussion

In this cohort, 70% of secundiparas (parity = 2) who underwent TOLAC subsequent to failed-VD achieved a VBAC. The main finding was the strong association of new-born BW differences between P1 and P2 with VBAC success. In general, when the BW of the new-born at P1 was higher than BW in the subsequent TOLAC (P2), then the chance of VBAC increased and vice versa; when the BW of the new-born at P1 was lower than the subsequent TOLAC, then the chance of failure increased. This association was shown to be strong with an OR of (9–11) for failed TOLAC, when the absolute weight difference was 400-500 g P2 > P1.

Attempting TOLAC is challenging for both the obstetrician and the parturient. A successful TOLAC is associated with an overall decreased risk of maternal morbidity including blood transfusion, hysterectomy, chorioamnionitis, postpartum haemorrhage, and injury to adjacent viscera compared to a planned CD; 3.1% versus 4.3% respectively. However, a failed TOLAC increases maternal morbidity significantly to an overall prevalence of 17% [15]. Therefore, identifying the appropriate candidates for TOLAC is crucial to minimise maternal risks, as such, decision-making for TOLAC should be individualised. As a result of the findings of the current study, when considering a TOLAC following a failed-VD, the BW of the new-born at the previous delivery is an important factor that should be taken into consideration.

It is well established that previous vaginal delivery significantly increases the success rate of TOLAC and is one of the strongest factors associated with VBAC. Thus, women at second delivery undergoing TOLAC, are a challenging, unique group [16–20]. Therefore, we neutralised the effect of previous vaginal delivery, as the current study only included secundiparas with one previous CD, excluding women with a previous vaginal delivery.

The limited data regarding this group of parturients is, further, reflected in the fact that both the RCOG Green top Guidelines: 'Birth after previous caesarean birth' and the ACOG Practice Bulletin: 'Vaginal birth after cesarean delivery' do not specifically address this group [16, 17]. Only two previous studies, with a of a total of 206 women, explored factors that could aid in decision making prior to attempting TOLAC following failed-VD (Table 3) [12, 13].

**Table 3:** Review of the literature; Trial of vaginal delivery subsequent to failed vacuum

**First author, year pub**	**Methodology**	**Study population**	**Number of patients**	**Outcome studied**	**Successful VD (%)**	**Factors from previous delivery found to be associated with a successful / failed TOLAC (OR)**
Jongen, 1998	Retrospective	Parity = 2	55 women had a TOLAC 41 had successful VBAC 14 and had failed	To determine the outcome of subsequent labor in primiparous women after a caesarean section for delay in descent in the second stage of labor in cephalic presentations with or without trial of instrumental vaginal delivery	75%	Not relevant to the study
Melamed, 2013	Historical prospective cohort	mixed parity 5% parity > 2 failed VE and following TOLAC in the same medical center	93 women had a TOLAC 57 had successful VBAC and 36 had failed	To assess the outcome of trial of labor after cesarean (TOLAC) in women with past failed operative vaginal delivery (OVD)	61%	**Associated with a successful TOLAC** POP position (OR = 3.3) **Associated with failed TOLAC** • prolonged 2^nd^ stage (OR = 3.4) • higher neonatal birthweight at P2 compared to P1 (OR = 13)
Levin, 2020	Historical prospective cohort	mixed parity 9% parity > 2 failed VE and following TOLAC in the same medical center	113 women had a TOLAC 76 had successful VBAC and 37 had failed	To examine trial of labor after cesarean delivery (TOLAC) success rates and its associated factors among women with a previous failed vacuum-assisted vaginal delivery	67%	None found
Reichman, Current study	Historical prospective cohort	Parity = 2 failed VE and following TOLAC including women who delivered elsewhere at P1	89 women had a TOLAC 62 had successful VBAC and 27 had failed	To examine success rates and its associated factors among primiparas with a previous cesarean due to failed vaginal delivery	70%	**Associated with a successful TOLAC** Lower neonatal birthweight at P2 compared to P1 absolute difference of 500 g (OR = 4.5) **Associated with failed TOLAC** • higher neonatal birthweight at P2 compared to P1, absolute difference of 500 g (OR = 11)

These studies showed similar VBAC rates of 61–67% to the 70% rate found in the current study [12, 13]. Only one of the two studies [12], revealed the strong association between the difference of new-born BW and VBAC with a similar OR as was found in the current study. It is possible that this association was not shown in Levin's study [13] as result of analysing the mean BW of new-borns, comparing P1 and P2 as two groups, and not comparing the detla for the BW in distinct categories and having each woman serve as her own control.

Unfortunately, other factors that could potentially affect TOLAC outcome, such as artificial reproductive treatments, pregnancy induced hypertension, gestation diabetes, POP and macrosomia, were rare among the study group, (less than 4%), and as such were not appropriate for statistical analysis. This low prevalence was also true for the two studies cited [12, 13].

A previous study found an association of HC with mode of delivery [21]. The same group found that HC had a stronger association to mode of delivery than birth weight [22]. In the current study, new-borns with HC difference larger than 10 mm at P2 compared to P1 were at risk for failed TOLAC (44.4% versus 28.6%), yet this finding didn’t reach statistical significance, possibly due to the small sample size.

The strengths of this study included: (1) The current study included 89 women, an increase of approximately 50% of women to published reports in the English literature. (2) Each woman served as her own control, and as such, much of the unknown confounding factors or known factors that were not available or missing from the dataset were neutralised. Examples of such factors included socioeconomic status, smoking and alcohol habits. (3) Powered studies of small samples have an advantage that if a factor is statistically significant, there is usually a strong association, emphasizing the clinical relevance. This was noted in the current study with a strong significant OR of 9–13 for failed TOLAC in cases where the BW of the new-born was higher at P2 than at P1.

The current study has some limitations. (1) Single-centre studies, by nature, are homogenous unlike multicentre studies that have potential differences in obstetric management and treatment protocols, which could affect the external validity. However, strict inclusion criteria of a narrowly defined group of parturients with similar characteristics described by others, minimises the significance of this limitation. (2) This study was based on actual birthweight of the new-born at P1 and P2, yet, from a practical point of view the birthweight at P2 is estimated prior to delivery by clinical evaluation or sonographically, both having known inaccuracies [23]. (3) The disadvantage of a small sample size leads to the possibility of the study being underpowered to show a significance of association with an OR of less than 4. For example, preterm delivery was documented in 2 (3%) women with a successful TOLAC, as compared to 3 (11%) with failed TOLAC, demonstrating a clinically significant ratio of 3.6, which was not statistically significant (*p* = 0.16). However, the sample size required to show a statistical significance with a two-sided *p* value ≤ 0.05 and a power of 80% would have been 366 women. Future "individual participant data meta-analysis", combining data of studies could potentially overcome this obstacle.

## Conclusion

Our study has shown that TOLAC subsequent to a failed-VD is a reasonable approach with a success rate of 70%. Women with an expected new-born BW similar or lower than the previous delivery have an increased likelihood of a VBAC. Secundiparas attempting TOLAC subsequent to a failed-VD should be counselled before delivery, taking into account the previous and the expected neonatal BWs, together with other known factors associated with VBAC as maternal BMI, Bishop score, Müllerian anomalies, maternal diabetes, and length of the inter-delivery interval [24]. In the era of individualised medicine aiming to base decisions on evidence-based medicine, there is need for additional studies addressing this issue.

## Data Availability

Date is available upon request from the corresponding author.

## Abbreviations

BW: Birthweight
CD: Caesarean delivery
EMR: Electronic Medical Record
Failed-VD: Failed vacuum delivery
HC: Head circumference
P1: Para 1
P2: Para 2
POP: Posterior occiput position
SZMC: Shaare Zedek Medical Centre
TOLAC: Trial of labour after caesarean
TOLAC failed-VD: Trial of labour after CD, secondary to a failed-VD in the previous delivery
VBAC: Vaginal birth after caesarean delivery
VE: Vacuum extraction
